# Quality and Microbiological Safety of Poultry Meat Marinated with the Use of Apple and Lemon Juice

**DOI:** 10.3390/ijerph20053850

**Published:** 2023-02-21

**Authors:** Anna Augustyńska-Prejsnar, Miroslava Kačániová, Małgorzata Ormian, Jadwiga Topczewska, Zofia Sokołowicz

**Affiliations:** 1 Department of Animal Production and Poultry Products Evaluation, Institute of Food Technology and Nutrition, University of Rzeszow, 35-959 Rzeszow, Poland; 2 Institute of Horticulture, Faculty of Horticulture and Landscape Engineering, Slovak University of Agriculture, 94976 Nitra, Slovakia

**Keywords:** microbiological quality, technological parameters, marinating, broiler chicken meat, apple juice, lemon juice

## Abstract

The aim of the study was to evaluate the use of apple juice for the marinating of poultry meat and its effect on the technological as well as sensory characteristics and microbiological safety of the raw product after heat treatment. Broiler chicken breast muscles were marinated for 12 h in apple juice (*n* = 30), a mixture of apple and lemon juice (*n* = 30) and compared with those in lemon juice (*n* = 30). The control group (*n* = 30) consisted of unmarinated breast muscles. Following the evaluation of the technological parameters (pH, L*, a*, b* colour, cutting force, cooking losses) quantitative and qualitative microbiological evaluations were performed on the raw and roasted products. The microbiological parameters were determined as total Mesophilic aerobic microorganisms, *Enterobacteriaceae* family, and *Pseudomonas* count. The bacterial identification was performed using a matrix-assisted laser desorption/ionisation time-of-flight mass spectrometry. The marinating resulted in lower pH value, but increased tenderness of raw and roasted products. Marinating chicken meat in both apple and lemon juices, including their mixtures and in the control sample, resulted in increased yellow saturation (b*). The highest flavour desirability and overall desirability were obtained in products marinated using a mixture of apple and lemon juice, while the most desirable aroma was obtained from products marinated with apple juice. A significant antimicrobial effect was observed in marinated meat products compared to unmarinated, irrespective of the type of marinade used. The lowest microbial reduction was observed in the roasted products. Apple juice can be used as a meat marinade because it promotes interesting sensory properties and improves the microbiological stability of poultry meat while maintaining the product’s good technological characteristics. It makes a good combination with the addition of lemon juice.

## 1. Introduction

Marinating is a traditional method of preparing meat for further processing or direct consumption, used in households, gastronomy, and the meat industry [1]. The marinating process allows the emphasising and highlighting of sensory features of poultry meat, such as taste, odour, colour and tenderness [2,3], it affects the technological features and effectiveness of the product and its safety, and extends the shelf life by limiting bacterial growth [4,5,6]. Poultry meat is becoming increasingly popular in the EU, including broiler chicken breast fillets, which are used most frequently to make marinated products that meet the convenience criteria ‘ready for thermal treatment food’ [7]. The convenience food of poultry origin is becoming more competitive than traditionally eaten meat and its products [8]. In addition, consumers are looking for meat products that meet the criteria for both convenience and functional food. The growth of poultry convenience food is caused by its expedient use, desirable sensory characteristics, and extended shelf life combined with high nutritional value [9]. However, interest in health-promoting food is related to the change in nutritional habits of consumers resulting from health care, which induces the search for inexpensive enriched meat products [10]. That is why both consumers and producers are increasingly leaning towards natural food additives which, in addition to flavour values, have a health-promoting effect [11]. The use of natural marinades or natural marinade additives have a decisive impact on the originality of a dish [12], and also on the taste and odour profile, as well as on its health-promoting effect [13]. In addition, natural products can replace synthetic additives and additional ingredients widely used in accordance with good manufacturing practice (GMP), such as ascorbic acid (E 300), sodium ascorbate (E 301); phosphoric acid salts, nitrites–potassium nitrite (E 249) and sodium nitrite (E 250) and nitrates–sodium nitrate (E 251) and potassium nitrate (E 252); citric acid (E 330), sodium citrate (E 331), which were placed on the list of food additives in the ‘meat’ category in the Commission Regulation (EU) 1129/2011 [14]. However, the most frequently used marinating additives in the food industry are sodium chloride and phosphates, which increase pH, water absorption, and tenderness of the marinated product [1,12]. The latest studies report an alternative natural marinating of meat using fruit juices as a process of sour marinating instead of synthetic ingredients [1,2,3,4,5,15,16,17]. Moreover, marinating in fruit juices may increase the diversity and range of value-added products in the poultry industry [12]. This has gained the interest of consumers as the operation is easy and non-time consuming while maintaining desirable food sensory properties [16].

Apples are one of the most often produced and consumed fruits in the world. Despite its availability, low price, and health-promoting properties, apple juice is not a very popular material for marinades [18]. Apple juice (100%) has a very good flavour, low calorific value, is composed of 12–14% carbohydrates, 0.1–0.3% protein, less than 0.1% lipids, and 0.3–1% organic acids (mainly α-malic acid, citric acid). It has a high content of biologically active substances, including vitamins (L-ascorbic acid 3–35 mg/100 g) and polyphenols. In apple juice, anthocyanidin, flavanol (also named flavan-3-ols), flavonols (mainly quercetin glycosides), and dihydrochalcones are a major subgroup of flavonoids [19]. According to Rajnić et al. [20] apple juice is a source of natural phenolic compounds with high antioxidant capacity. The high health-promoting value of apple juice is a result of the content of an insoluble fraction of nutritional cellulose, which is a major component of a healthy diet. Apple juice is a rich source of macroelements, such as phosphorus and potassium, and in a lesser content also of magnesium, calcium, and zinc [19]. Variability of biochemical content of apple juice depends on variety, ripeness, and climatic conditions of fruit growth.

Lemon juice (*Citrus limon*) is a widely used ingredient of meat marinades, most often used as a preservative and improver of sensory properties of products [21,22]. It is a rich source of L-ascorbic acid (0.51–0.53 mg/100 g), and also B-type vitamins, Beta carotene, macro and microelements. It also contains citric acid (5–10%), cellulose (3%), sugars (2–3%), and proteins (1%). Biological activity of *Citrus limon* juice is a result of a high content of polyphenolic compounds, of which a major part are flavonoids: apigenin, diosmin, eriodictyol, hesperidin, quercetin, naringenin; flavonols: isorhamnetin, quercetin, rutoside; and flavanones: hesperidin, naringenin and phenolic acids (ferulic and sinapinic) [23]. Citrous flavonoids have antioxidant and anti-inflammatory properties and play an important part in prevention of diseases related to stress and inflammatory conditions of the organism [24]. The substances contained in lemon juice have a very high antibacterial and antimicrobial activity [25].

Microbiological evaluation, including bacterial identification, is of key importance in assessing the safety and quality of marinated products, particularly of natural origin [26,27]. The method, based on the analysis of organisms’ protein profile (Maldi TOF MS) [28], is recognized in food microbiology as being quick, inexpensive, and very accurate in identification of bacteria [29]. There are studies that focused on the effects of marinades on the microbiological quality and safety of poultry meat [30,31,32], but not many are focused on marinated chicken meat [6,33,34] as well as marinades, which contain organic acids as active substances against microorganisms [4,35,36,37]. The stability of the meat and meat product is influenced by the type of marinade used [38,39] or the treatment and storage conditions [40,41,42]. There are also some studies [6] on the effect of marinating conditions (marinating temperature and time) on the microbial succession and physicochemical profile of chicken meat.

The aim of the study was an assessment of apple juice in marinating pectoral muscles of broiler chickens. Verification included the impact of marinating with 100% pressed apple juice, a mixture of apple juice and lemon juice, and with lemon juice (for comparative reasons) on the technological and sensory properties and microbiological safety of raw product and thermally-treated product.

## 2. Materials and Methods

### 2.1. Raw Material for the Study

The raw material for the study was breast muscles (musculus pectoralis) from 25-day-old ROSS 308 broiler chickens kept in the same production cycle from one chicken producer. The raw material was obtained from a local abattoir located in the Podkarpackie Voivodeship (Rzeszow, Poland). The mechanical slaughter of broiler chickens was carried out in accordance with industry standards specified for poultry abattoirs in the EU, after previous veterinary examination [43]. The carcasses were cooled in two stages, in water at 16 °C, and then using the air stream method to obtain the meat temperature of 2 °C. The carcasses were partitioned mechanically, and the breast muscles were cut out manually. Individual breast muscles (*n* = 120, average weight 180 ± 50 g), were taken to the laboratory in isothermal containers and kept in a refrigerator at 4 °C for 2 h. The meat samples (individual breast muscles) were divided into two groups: non-marinated (*n* = 30) and marinated (*n* = 90) and weighed with 0.01 g accuracy and marked individually. The non-marinated samples (control group) were kept at 4 °C in containers for meat storage.

### 2.2. Marinating Procedure and Sample Cooking

The study uses three types of sour marinades; namely, apple juice (group AJ), the mixture of apple and lemon juices (group AJL), and lemon juice as a comparison (group LJ). The concentration of all marinades was specified in relation to the average apple juice pH (3.44 ± 0.02). In the group AJ, the marinade was 100% pressed apple juice from ecological grown apples in the ‘Sady Wincenta 100% Natury’ farm in Poland. This was a natural, turbid juice. According to the producer’s declaration, the calorific value was 51 kcal per 100 mL, the content of available carbohydrates 12.76 g, total sugars 11.13 g, total fat 0.10 g including saturated fatty acids 0.10 g, nutritional cellulose 0.15 g, protein 0.20 g, total ash 0.16 g, and sodium 0.30 mg. The lemon marinade (group LJ) was made from lemon juice (pH 2.60 ± 0.01) and cooled boiled water (pH 8.01). The lemons were BIO 100%, ‘Dary Natury’ brand, Poland, PL-EKO-01-001493, country of origin: Italy. According to the producer’s declaration, the calorific value of the juice was 129 kJ/30 kcal; carbohydrates 7.1 g; including sugars 1.9 g; protein 0.5 g; salt 0.1 g, vitamin C 50 mg (RWS 62.5%). The mixture of apple and lemon juices (group AJL) was set at 1:1, using such amount of lemon juice as to keep the marinade pH at 3.44. The marinades were prepared in glass vessels, then carefully mixed with a glass rod and cooled down to 5 °C ± 1 °C. The prepared marinating samples (*n* = 90) were randomly assigned to groups AJ, AJL, and LJ (*n* = 30 in each group). The marinating method was static, involving pouring the marinade on the meat and soaking it in the marinade in a 1:1 proportion. The marinating process was conducted at 4 °C in containers safe for food, while the marinated samples were taken for examination after 12 h. Prior to and after the marinating process, the samples were weighed with 0.01 g accuracy (Ohaus V1193 scale, Parsippany, NJ, USA) and individually marked. The control group–C (*n* = 30) included single non-marinated breast muscles. The non-marinated and marinated muscles were weighed with 0.01 g accuracy and were thermally treated (180 °C) in a steam convection oven, fitted with an integrated thermal probe (AEG BSK792320M, Berlin, Germany) to ensure the attainment of 78 °C ± 2 °C inside the meat sample

### 2.3. Quality Parameters

#### 2.3.1. Assessment of Technological Characteristics

In order to determine the marinade absorption, the samples of marinated and non-marinated meat were weighed before (M_1_) and after (M_2_) marinating. The marinade absorption was calculated according to the formula: marinade absorption (%) = [(M_2_ − M_1_)/M_1_] × 100 according to the Yunga and Buhr method [44]. The technological parameters before and after the marinating were measured in both the raw products and products after roasting in all samples from groups AJ, AKL, and LJ. The active acidity (pH) of the products was measured using an HI 99,163 pH-meter (Hanna Instrument Company, Woonsocket, RI, USA), equipped with an FC232 electrode (Hanna Instrument Company, Woonsocket, RI, USA). Before the measurements, the pH-meter was calibrated in buffers with pH 4.01 and pH 7.01 (Hanna Instrument Company, Salaj, Romania). Six measurements were taken for each sample, maintaining the same procedures. The colour assessment of the samples’ cross section was conducted using the reflection method with a Chrome Meter colorimeter (Konica Minolta, Osaka, Japan), equipped with a CR 400 head (ø = 11 mm). The colorimeter was, prior to the measurement, calibrated against a standard (No. 21833042). The reflectance method was applied at the D65 standard lighting and 2o observer. The measurements results were read in the colorimetric system CIE LAB (CIE 1978)—L* (lightness), a* (redness) and b* (yellowness), taking three measurements for each sample [45]. The tenderness was measured based on the shear force (Fmax), using a Zwick/Roell BT1-FR1.OTH.D14 machine (Zwick GmbH & Co., KG, Ulm, Germany) with a Warner-Bratzler knife (V-blade). Samples of 100 mm^2^ in area and 50 mm in length cut along the muscle fibres were prepared before the measurements [46]. The cuts were made at a blade speed of 100 mm·min^−1^ and 0.2 N, until the samples were fully cut (min. 3 repetitions from each trial). The cutting measurement results were elaborated using the Test Xpert II software, ver.3.1 [47]. The cooking losses were determined when the samples were cooled down to room temperature based on the weight loss, measured prior to and after the thermal treatment.

#### 2.3.2. Sensory Assessment

The sensory assessment of both the marinated and non-marinated samples of roasted products was conducted using the scaling method according to the methodology developed by Baryłko-Pikielna and Matuszewska [48]). Special assessment sheets were developed, with a 5-point evaluation with a defined value limit, including the following qualitative indices: odour intensity (very negative—typical, very strong), flavour intensity (very negative, very sour—typical, very desirable), odour desirability (not desirable—highly desirable), flavour desirability (not desirable—highly desirable), juiciness (very dry—very juicy), tenderness (very hard–very tender), and general desirability (undesirable—desirable). The assessment was performed by a team of 10 women aged between 29–62, with a verified sensory sensitivity according to ISO 8586-2 [49], and at least 4 years of experience in the evaluation of meat and meat products. For the correct assessment, the samples were cut, coded, and presented to the panellists in white dishes. The sets of samples for individual evaluators were presented in a specific order which was changed during the second assessment session to prevent a possible impact of the previous trial on the successive one. The panellists took breaks (30 s) between each test and rinsed their mouths with mineral water. The assessment was conducted in a room devoid of odours, at 20 °C ± 2 °C, in accordance with the standard [50].

#### 2.3.3. Microbiological Analyses

Breast muscles [10 g] were used for the evaluation, using sterile instruments for all samples in the study. Having been put in a sterile stomacher bag, the samples were subjected to homogenization using a 0.1% peptone 90 mL water (pH = 7.0) with dilutions ranging from 10^−1^ to 10^−3^ at 20 °C for 30 min. The determination of total number of mesophilic aerobic microorganisms (MAM), which is necessary for calculation of the parameters which determine colony-forming units per gram of sample [cfu/g], was performed on samples cultured on Plate count agar (PCA, Biocorp, Issoire, France). Inoculated samples were incubated in aerobic conditions at 37 °C for 24 h. With respect to Pseudomonas spp., however, a medium for isolation of Pseudomonas agar (PA, Biocarp, Issoire, France) was applied. Aerobic incubation of the samples at 25 °C over a 48 h period was conducted, using Violet Red Bile Glucose Agar (VRBL, Biocorp, Issoire, France) in order to isolate *Enterobacteriaceae* family. The VRBL agar plates were incubated at 37 °C over 24 h. The analysis was performed in triplicate [26].

#### 2.3.4. Mass Spectrometry in Isolates Identification

The analytical samples for MALDI-TOF MS Biotyper for trials analysis were prepared in line with the extraction procedure provided by Bruker Daltonik, Bremen, Germany. The bacterial colony suspended in 300 μL water (Sigma-Aldrich, St. Louis, MO, USA) and 900 μL absolute ethanol (Bruker Daltonik, Bremen, Germany) was mixed and centrifuged ten times at 13,000 rpm for 2 min. Having rejected the supernatant, the pellets were then centrifuged several times. Next, the pellets devoid of supernatant were mixed with 10 μL 70% formic acid (*v*/*v*) (Bruker Daltonik, Bremen, Germany) and centrifuged repeatedly [26]. A polished steel target plate was stained with 1 μL of the supernatant and finally air-dried at room temperature. An amount of 1 μL MALDI matrix (saturated solution of α-cyano-4-hydroxycinnamic acid, HCCA, Bruker Daltonik, Bremen, Germany) consisting of 50% acetonitrile and 2.5% trifluoroacetic acid (Sigma Aldrich, St. Louis, MO, USA) was applied to each sample. Next, the Microflex LT MALDI-TOF mass spectrometer (Bruker Daltonik, Bremen, Germany), that operates in a linearly positive mode with a mass range of 2000–20,000 Da was used to automatically generate mass spectacles. The device calibration was achieved based on the Bruker bacterial standard. The MALDI Biotyper 3.0 software (Bruker Daltonik, Bremen, Germany) was used for processing spectrometric data and to obtain the results. The identification criteria applied included score ranges of 2300–3000 for highly probable identification at the species level; 2000–2299 for safe genus identification with probable species identification; and 1700–1999 for probable identification at the genus level [35].

### 2.4. Statistical Analysis

The statistical analysis of the results was conducted using the analysis of variance (ANOVA) and the Statistica 13.3 software package [51]. Having determined the mean ($\bar{x}$) and standard deviation (s), the data were verified for normality using the Kolmogorov–Smirnov test. The homogeneity of variance was verified using the Brown-Forsythe test. The Tukey’s test was used to indicate the significance of differences between the means in the groups at the 95% (α = 0.05) confidence level. The differences were considered significant if *p* ≤ 0.05.

## 3. Results and Discussion

The percentage of marinade absorption significantly (*p* < 0.05) differed according to the marinade used (Table 1). It was observed that marinade absorption was significantly higher in products marinated with lemon juice and a mixture of apple and lemon juice. This could be explained by swelling and increased extraction of myofibrillar proteins, due to its increase in ionic strength [52]. Unal et al. [5] indicated that chicken breast muscles marinated in lemon juice had higher marinade absorption compared to products marinated in grapefruit juice and citric acid. Kumor et al. [22] obtained similar results of increased marinade absorption, using lemon juice to marinate the muscles of the hen breast after laying, compared to the unmarinated product., Likewise, turkey breast muscles marinated with grapefruit juice and citric acid [53] as well as pork loin marinated cherry and plum juice [1] yielded similar results.

The active acidity of marinated product is closely related to the acidity of the marinade and its organic acid content [5,22,53,54]. This was confirmed in the current study. In all groups of marinated products, the pH of the tested products was significantly lower than that of the unmarinated meat (Table 1). However, the greatest decrease (pH = 0.04) was observed in the raw product marinated in lemon juice marinades, corroborating existing literature [22,53]. Similar results were obtained using apple and plum juice [21].

An important factor affecting the technological quality of marinated meat products is its colour [35]. The study showed that the application of lemon juice to marinades (group LJ and ALJ) significantly (*p* ≤ 0.05) affected the lightening (increase in parameter L*, a decrease in red saturation a*) of raw and roasted marinated products compared to the product marinated in apple juice (group AJ) and meat not subjected to the marinating process (Table 1). The resulting colour is due to the pigment and acidity of the fruit juices used for marinating. One possible reason for the increase in L* value is swelling of muscle proteins and a change in light reflection at low pH and ionic strength, resulting in a lighter colour [1,5,35]. According to Strzyżewski [55], a reduction in the pH value of meat results in an increase in its lightness of colour. Previous studies [35,56,57] showed that the use of lemon juice marinades contributed to the lightening of the marinated product. Unal et al. [5] and Serdaroğlu et al. [53] obtained similar results by conducting studies on chicken and turkey meat marinated using lemon and grapefruit juices. In our study, the use of apple juice for marinating (group AJ and ALJ) increased (*p* ≤ 0.05) the intensity of the colour to yellow (b*), compared to marinating using lemon juice (group LJ) and the control sample.

Several studies [53,56,58] suggest that the use of sour fruit juices as a marinade for meat can beneficially improve the textural characteristics of meat products. Meat tenderness is an important characteristic that determines the quality and consumer acceptance of a marinated product. Our own research indicates a significant (*p* ≤ 0.05) change in the mechanical properties of marinated products compared to raw unmarinated material (Table 1). Within the marinated products, the raw products marinated with lemon juice marinade were characterized by the lowest cutting force, using the Warner-Bratzler cutter (best tenderness). However, following the roasting, the tenderness of all marinated products remanded unchanged and was better than the unmarinated product. According to Burke and Monahan [59], a proposed mechanism for the softening effect of acid marinades is that muscle fibers swell and connective tissue dilutes the amount of resistant material so that tenderness and swelling reach a maximum under the same conditions. Unal et al. [5] obtained a beneficial effect to hardening of broiler chickens breast muscles following a 24 h treatment with lemon juice compared to results obtained using citric acid, grapefruit juice, and in the control group. Kumar et al. [60] using 20% lemon juice obtained favourable tenderness compared to marinating with ginger extract.

In our study, there were no significant differences (*p* > 0.05) in cooking losses of products marinated in apple juice, a mixture of apple and lemon juice as well as in diluted lemon juice (Table 1). As studies Obuz and Cesur [61] and Serdaroğlu et al. [53] indicate, treating within acidic marinades, including lemon juice, can increase cooking losses of marinated products. Erge et al. [12] showed lower cooking losses in apple juice compared to plum juice, indicating that apple juice may be an alternative marinade in terms of cooking losses.

Sensory evaluation is a commonly used method in the evaluation of marinated meat products because of its quick and simple information on the characteristics that determine the consumer’s choice of the product [62,63]. It provides a valuable source of data on consumer perceptions of both the product as a whole and its individual characteristics [5]. The studies conducted confirmed the beneficial effects of marinating with apple and lemon juice on the sensory attributes of the roast product (Table 2). The study showed that marinating in apple juice and lemon juice significantly (*p* ≤ 0.05) improved the desirability of the taste, juiciness, and tenderness of the products compared to the control group. The confirmation of the use of acidic fruit juices to improve sensory characteristics, mainly by reducing the hardness and improving the tenderness of the product, has been reflected in a number of works [5,22,53,57,61]. However, sour fruit marinades tend to cause a taste change towards a sour, foreign flavour [61], which was not confirmed in our study. Across the research groups, the highest taste desirability and overall desirability was obtained in the poultry product marinated in a mixture of apple and lemon juice (group ALJ), while the most desirable taste (intensity and desirability) was obtained in the product marinated in apple juice (group AJ). Erge et al. [12], using apple juice concentrate to marinate chicken breast fillets obtained a product with high sensory acceptability. Another study by Unal et al. [5] found that chicken breast meat marinated in lemon juice had higher juiciness and lower hardness, but was inferior in overall appearance compared to meat marinated in grapefruit juice and citric acid. On the contrary, Lytou et al. [6], showed that a marinade in which lemon juice and pomegranate juice were combined resulted in a reduction in microbial numbers and led to desirable sensory characteristics. As reported by Raba et al. [18], a pork product marinated/injected with apple juice (20%) and apple cider (20%) improved sensory qualities and was accepted by the evaluation panel as a natural meat tenderizer. The authors reported that the main sensory attributes of apple juice were a pleasant aroma, an optimal balance between sweetness and acidity, colour, and texture.

The effect of marinades on the indigenous microbiota of broiler breast muscles is presented in Table 3. Some microbiological analyses of marinated chicken breast meat were evaluated in our study. The number of mesophile aerobic microorganisms was between 0.00 in roasted samples products marinated with apple, mixture of apple–lemon, and lemon juice, to 2.60 log cfu/g in raw samples products marinated with apple juice. The results showed that *Enterobacteriaceae* family numbers ranged from 0.00 in roasted samples of marinated meat products with apple, mixture of apple–lemon, and lemon juice, to 1.63 log cfu/g in raw samples of muscles marinated in lemon juice. The number of Pseudomonas ranged from 0.00 in raw samples products marinated with a mixture of apple as well as lemon juices and roasted samples products marinated with apple, mixture of apple and lemon, and lemon juice, to 1.85 log cfu/g in raw samples of muscles marinated in lemon juice.

The results achieved in the current study showed that the initial microbiota of fresh chicken meat is in line with the published data [64,65,66]; however, there are also few studies focused on the effect of marination on the indigenous microbiota of chicken meat [67]. There was a significant antimicrobial effect of marination in this study that resulted in reduced microbial population regardless of the type of marination. The lowest microbial reduction within the different members of the association was observed in roasted AJ, ACJ, and CJ samples compared to raw AJ, ACJ, and CJ samples (Table 4). Previous studies have reported similar reductions (1–3 log units) in TVC in comparison with the current work following the treatment of chicken meat with organic acids at similar concentrations [64,68,69] due to the separate investigation of the enumerated microbial groups. Furthermore, the population of *Pseudomonas* spp. After marination in lemon juice (4.6% citric acid) was in line with Bolton et al. [64], who reported a reduction of up to 2.6 log cfu/cm^2^ after treatment in 5% citric acid. Similar results with pomegranate marination (1.8% citric acid) were reported by Del Rio et al. [68], who observed 1.4 log unit decrease after treatment in 2% citric acid. Pomegranate juice contains polyphenols (such as tannins and flavonoids) and anthocyanins with potential antimicrobial activity [70,71,72]. *Enterobacteriaceae* are one of the potential bacterial spoilage groups in poultry meat. However, the involvement of these bacteria and their role in poultry meat spoilage has not been fully investigated. Results of marinate treatments that have effectively inhibited coliform growth are available in literature [73].

**Figure 1:**
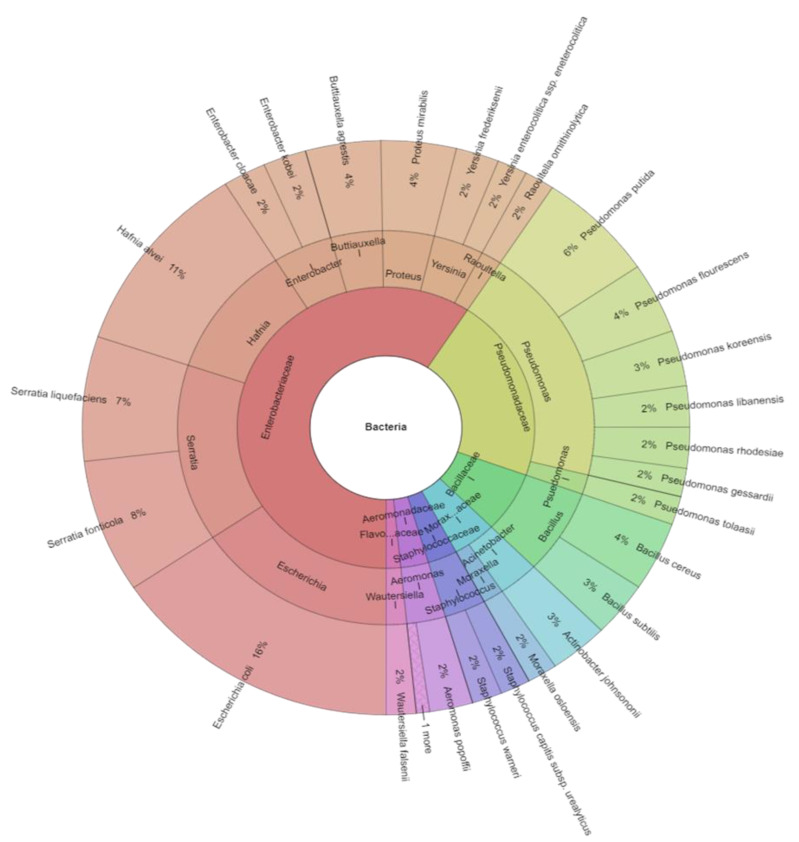
Krona chart: Isolated species of bacteria from marinated raw products.

**Figure 2:**
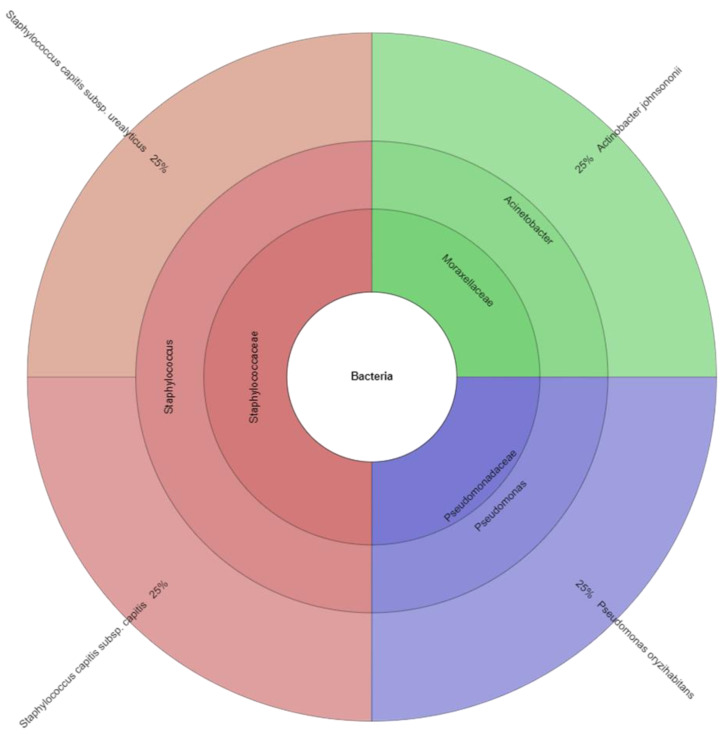
Krona chart: Isolated species of bacteria from marinated roasted products.

**Figure 3:**
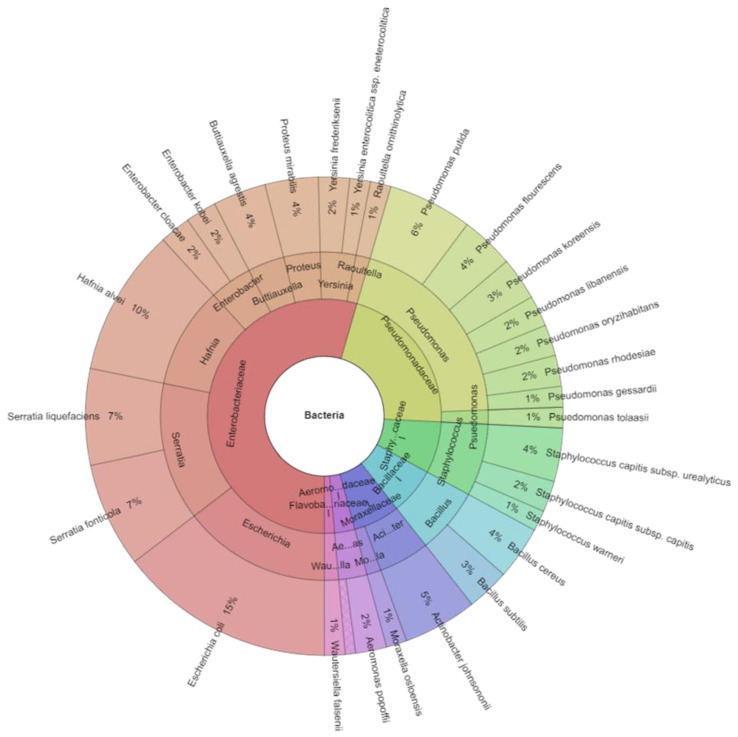
Krona chart: Isolated species of bacteria from raw and roasted chicken’s meat.

A total of 140 isolates were identified with mass spectrometry (Table 4). The results of microbial identification obtained using a MALDI-TOF MS Biotyper are shown in Figure 1, Figure 2 and Figure 3. The presented results had a score value ≥2.00. The identifications were made for 26 bacterial samples isolated from broiler chicken’s meat (group C); 37 bacterial samples isolated from marinated raw products (group AJ), 21 (group ALJ), 44 (group LJ) as well as 12 bacterial samples from marinated roasted products, of which 78.65% were correctly identified. A total of 128 isolates were identified using MALDI-TOF MS Biotyper in raw broiler chicken’s meat and 12 isolates from roasted boiler chicken’s samples from the control group. There were also 7 families (*Aeromonadaceae*, *Bacillaceae*, *Enterobacteriaceae*, *Flavobacteriaceae*, *Moraxellaceae*, *Pseudomonadaceae*, *Staphylococcaceae*), 15 genera and 29 species isolated from all samples. An amount of 7 families, 15 genera and 27 species were isolated in raw products, while 3 family, 3 genera and 4 species in roasted products were isolated. The most commonly isolated species were *Escherichia coli* (20 isolates), *Hafnia alvei* (14 isolates), *Serratia fonticola* (10 isolates), and *Serratia liquefaciens* (9 isolates). The most commonly isolated species came from *Enterobacteriaceae* family, together 11 species, including *B. agrestis*, *E. cloacae*, *E. kobei*, *E. coli*, *H. alvei*, *P. mirabilis*, *R. ornithinolytica*, *S. fonticola*, *S. liquefaciens*, *Y. enterocolitica*, and *Y. frederiksenii*. The second most isolated family was Pseudomonadaceae, where the most isolated genera was *Pseudomonas* with eight species including *P. flourescens*, *P. gessardii, P. koreensis*, *P. libanensis*, *P. oryzihabitans*, *P. putida*, *P. rhodesiae*, and *P. tolaasii*.

The microbiota of chicken breast and thigh fillets at the end of storage was dominated by Proteobacteria and followed by Firmicutes [74], which is similar to findings of the current study. Genera *Acinetobacter*, *Photobacterium*, *Pseudomonas*, and members of Vibrionaceae family were found in raw meat. *Pseudomonas* was recognized as a predominant psychotropic meat spoiler [75,76,77,78,79,80,81]. *Pseudomonas* were found only at high abundances (33.0%) on fresh thigh fillets. *Acinetobacter*, which belongs to the Moraxellaceae family, was detected on most chicken samples despite the observed differences in detection abundances. Although it is a strictly aerobic, gram-negative microorganism, its occurrence on fresh and spoiled meats under aerobic, modified atmosphere, and vacuum-storage conditions is well documented [75,78,82]. The most isolated species of gram-negative bacteria (24 isolates) and gram-positive bacteria had only five isolates in the current study. It was considered that gram-negative bacteria are more sensitive to acidic conditions than gram-negative bacteria [83]. Other types of microorganisms such as *Lactobacillus algidus*, *Lactobacillus sakei*, *Leuconostoc mesenteroides*, *Leuconostoc carnosum*, *Carnobacterium maltaromaticum*, *Carnobacterium divergens*, *Brochothrix thermosphacta* and *Serratia proteamaculans* were isolated by Björkroth [84] from marinated meat. *Pseudomonas* spp., *Enterobacteriaceae*, *Staphylococcus aureus*, *Salmonella* spp. and other microorganisms cause spoilage in marinated poultry [85]. It has been suggested [86] that the inhibiting effect of lactic acid on *Staphylococcus aureus* is mainly due to the low pH. Marinade solutions containing lactic acid or sodium lactate [85] have been used to suppress the growth of *Enterobacteriaceae*.

## 4. Conclusions

Marinating with apple juice, a mixture of apple and lemon juice, and lemon juice resulted in a reduction in pH value, increased tenderness of raw and roasted products, and improved microbiological quality. Regardless of the type of marinade, the lowest microbial reduction occurred in roasted products. The mixture of apple and lemon juice was shown to be more effective in reducing the number of aerobic mesophilic bacteria and *Pseudomonas* spp. in marinated raw products.

Both products, raw and roasted, marinated in apple juice and in a mixture of apple and lemon juice were characterised by higher colour saturation in the yellow direction, compared to those marinated in lemon juice and unmarinated. Marinating broiler chicken meat in a mixture of apple and lemon juice can be used to improve the flavour and overall desirability of the product. For the most desirable aromatic bouquet, marinating in apple juice is most beneficial.

Using apple juice to marinate poultry meat is an interesting alternative to the commonly used lemon juice marinade. Increasing the variety and range of microbiologically safe convenience products made from poultry meat can make a difference in global communities.

The results obtained indicate the possibility of using apple juice in the meat industry and are exploratory in terms of the composition of fruit marinades for meat. They provide a basis for further research into the use of apple juice in the processing of poultry meat.

## Figures and Tables

**Table 1:** Impacts of fruit juice marinating on the technological characteristics of marinated products.

Parameter	Not Marinated Group C	After Marinating			
		Group AJ	Group ALJ	Group LJ	*p*-Value
**Raw Products**					
Marinade uptake (%)	-	0.47 ^b^ ± 0.34	0.96 ^a^ ± 0.28	1.06 ^a^ ± 0.30	**0.006**
pH	6.06 ^a^ ± 0.09	5.85 ^b^ ± 0.08	5.83 ^b^ ± 0.09	5.88 ^c^ ± 0.04	**0.0000**
Colour:					
L*—lightness	53.24 ^a^ ± 3.33	54.18 ^a^ ± 3.20	56.43 ^b^ ± 2.02	59.84 ^b^ ± 3.09	**0.0001**
a*—redness	3.96 ^a^ ± 0.40	2.89 ^b^ ± 0.61	3.03 ^b^ ± 0.70	1.09 ^c^ ± 0.24	**0.0000**
b*—yellowness	2.43 ^a^ ± 0.84	5.63 ^b^ ± 0.50	4.98 ^b^ ± 0.72	3.84 ^c^ ± 0.38	**0.0000**
Shear force (N)	29.02 ^a^ ± 4.10	24.14 ^a^ ± 3.18	23.58 ^b^ ± 3.83	16.28 ^c^ ± 2.95	**0.0001**
**Roast Products**					
pH	6.24 ^a^ ± 0.20	6.04 ^b^ ± 0.10	6.01 ^b^ ± 0.10	5.95 ^b^ ± 0.14	**0.015**
Cooking losses (%)	34.06 ± 3.10	33.64 ± 2.89	34.94 ± 3.60	34.20 ± 2.78	0.1132
Colour:					
L*—lightness	82.96 ^a^ ± 2.23	83.35 ^a^ ± 1.66	86.20 ^b^ ± 0.85	87.58 ^b^ ± 1.57	**0.0000**
a*—redness	5.83 ± 0.68	5.32 ± 0.64	5.55 ± 0.28	5.86 ± 0.35	0.1727
b*—yellowness	7.58 ^a^ ± 1.20	10.08 ^c^ ± 2.45	9.14 ^c^ ± 0.32	5.39 ^b^ ± 0.66	**0.0000**
Shear force (N)	26.12 ^a^ ± 3.65	18.83 ^b^ ± 3.37	17.02 ^b^ ± 3.64	16.88 ^b^ ± 3.01	**0.0004**

Explanations: group C (control)—broiler breast muscles not marinated (*n* = 10); group AJ—broiler breast muscles marinated with apple juice (*n* = 10); group ALJ—broiler breast muscles marinated with a mixture of apple and lemon juices (*n* = 10); group LJ—broiler breast muscles marinated in lemon juice (*n* = 10); a, b, c—values in rows with different letters differ significantly *p* ≤ 0.05; *p*-Valu*e* in bold significant.

**Table 2:** Impacts of fruit juice marinating on the sensory assessment of marinated roasted products (points).

Parameters	Group				
	C	AJ	ALJ	LJ	*p*-Value
Odour intensity	3.92 ^a^ ± 0.58	4.83 ^b^ ± 0.26	4.67 ^bc^ ± 0.41	4.00 ^ac^ ± 0.35	**0.0023**
Flavour intensity	3.75 ^a^ ± 0.42	4.83 ^b^ ± 0.26	4.75 ^bc^ ± 0.27	4.20 ^ac^ ± 0.57	**0.0003**
Odour desirability	4.08 ± 0.20	4.50 ± 0.45	4.33 ± 0.26	4.30 ± 0.45	0.2658
Flavour desirability	3.08 ^a^ ± 0.38	4.17 ^b^ ± 0.52	4.92 ^c^ ± 0.20	4.10 ^b^ ± 0.22	**0.0000**
Juiciness	3.08 ^a^ ± 0.32	4.25 ^b^ ± 0.52	4.17 ^b^ ± 0.41	4.10 ^b^ ± 0.22	**0.0004**
Tenderness	3.70 ^a^ ± 0.52	4.25 ^b^ ± 0.27	4.58 ^b^ ± 0.49	4.60 ^b^ ± 0.57	**0.0011**
External color	4.08 ± 0.38	4.25 ± 0.27	4.33 ± 0.26	4.20 ± 0.57	0.7149
Section color	3.92 ± 0.38	4.00 ± 0.32	4.25 ± 0.27	4.10 ± 0.22	0.3003
General desirability	3.67 ^a^ ± 0.26	4.33 ^b^ ± 0.26	4.62 ^c^ ± 0.38	4.20 ^b^ ± 0.27	**0.0012**

Explanations: group C (control)—broiler breast muscles not marinated (*n* = 10); group AJ—broiler breast muscles marinated with apple juice (*n* = 10); group ALJ—broiler breast muscles marinated with a mixture of apple and lemon juices (*n* = 10); group LJ—broiler breast muscles marinated using lemon juice (*n* = 10); values a, b, c in rows with different letters differ significantly *p* ≤ 0.05; *p*-Value in bold significant.

**Table 3:** The effect of fruit juice marinating on microbiological parameters of marinated products [log cfu/g].

Parameter	Not Marinated Group C	After Marinating			
		Group AJ	Group ALJ	Group LJ	*p*-Value
**Raw products**					
MAM log cfu/g	2.36 ^a^ ± 0.26	2.45 ^a^ ± 0.32	1.12 ^b^ ± 0.21	2.60 ^a^ ± 0.32	**0.0007**
EF log cfu/g	0.32 ^a^ ± 0.27	0.25 ± 0.15 ^b^	0.28 ^b^ ± 0.28	1.63 ^c^ ± 0.29	**0.0004**
PC log cfu/g	0.78 ^a^ ± 0.18	1.08 ^b^ ± 0.24	nd	1.85 ^c^ ± 0.31	**0.0000**
**Roast products**					
MAM log cfu/g	0.30 ± 0.22	nd	nd	nd	-
EF log cfu/g	0.34 ± 0.25	nd	nd	nd	-
PC log cfu/g	1.15 ± 0.26	nd	nd	nd	-

Explanations: group C (control)—broiler breast muscles not marinated (*n* = 10); group AJ—broiler breast muscles marinated with apple juice (*n* = 10); group ALJ—broiler breast muscles marinated with a mixture of apple and lemon juices (*n* = 10); group LJ—broiler breast muscles marinated in lemon juice (*n* = 10); MAM—mesophile aerobic microorganisms; EF—*Enterobacteriaceae* family; PC—*Pseudomonas* count; nd—not detected; a, b, c—values in rows with different letters differ significantly *p* ≤ 0.05; *p*-Value in bold significant.

**Table 4:** Isolated bacteria from marinated raw and roasted products.

Isolated Species/Experimental Group	Raw Products				Roast Products	Total Isolates
	Group				Group	
	C	AJ	ALJ	LJ	C	
*Actinobacter johnsononii*			4		3	7
*Aeromonas encheleja*		1				1
*Aeromonas popoffii*	3					3
*Bacillus cereus*	2	3				5
*Bacillus subtilis*		4				4
*Buttiauxella agrestis*		2		3		5
*Enterobacter cloacae*		3				3
*Enterobacter kobei*				3		3
*Escherichia coli*	5	4	5	6		20
*Hafnia alvei*	3	3	4	4		14
*Moraxella osloensis*	2					2
*Proteus mirabilis*				5		5
*Pseudomonas flourescens*		2		3		5
*Pseudomonas gessardii*		2				2
*Pseudomonas koreensis*				4		4
*Pseudomonas libanensis*		3				3
*Pseudomonas oryzihabitans*					3	3
*Pseudomonas putida*	4			4		8
*Pseudomonas rhodesiae*				3		3
*Psuedomonas tolaasii*	2					2
*Raoultella ornithinolytica*		2				2
*Serratia fonticola*	3		3	4		10
*Serratia liquefaciens*		2	2	5		9
*Staphylococcus capitis* subsp. *capitis*					3	3
*Staphylococcus capitis* subsp. *urealyticus*		2			3	5
*Staphylococcus warneri*	2					2
*Wautersiella falsenii*		2				2
*Yersinia enterocolitica* subsp*. eneterocolitica*		2				2
*Yersinia frederiksenii*			3			3
Total isolates	26	37	21	44	12	140

Explanations: group C (control)—broiler breast muscles not marinated (*n* = 10); group AJ—broiler breast muscles marinated with apple juice (*n* = 10); group ALJ—broiler breast muscles marinated with a mixture of apple and lemon juices (*n* = 10); group LJ—broiler breast muscles marinated in lemon juice (*n* = 10).

## Data Availability

The data that support the findings of this study are available from the corresponding author (A.A.-P.) upon reasonable request.
